# Secretory IgA is Concentrated in the Outer Layer of Colonic Mucus along with Gut Bacteria

**DOI:** 10.3390/pathogens3020390

**Published:** 2014-04-29

**Authors:** Eric W. Rogier, Aubrey L. Frantz, Maria E. C. Bruno, Charlotte S. Kaetzel

**Affiliations:** 1 Department of Microbiology, Immunology, and Molecular Genetics, University of Kentucky, Lexington, KY 40536, USA; E-Mails: erogier@cdc.gov (E.W.R.); aubrey.frantz@unt.edu (A.L.F.); mebrun2@uky.edu (M.E.C.B.); 2 Current address: Centers for Disease Control and Prevention, Division of Parasitic Diseases and Malaria, Malaria Branch, Atlanta, GA 30333, USA; 3 Current address: Division of Liberal Arts & Life Sciences, University of North Texas at Dallas, Dallas, TX 75241, USA

**Keywords:** intestinal epithelium, gut bacteria, secretory IgA, polymeric immunoglobulin receptor (pIgR), intestinal mucus, mucin-2

## Abstract

Antibodies of the secretory IgA (SIgA) class comprise the first line of antigen-specific immune defense, preventing access of commensal and pathogenic microorganisms and their secreted products into the body proper. In addition to preventing infection, SIgA shapes the composition of the gut microbiome. SIgA is transported across intestinal epithelial cells into gut secretions by the polymeric immunoglobulin receptor (pIgR). The epithelial surface is protected by a thick network of mucus, which is composed of a dense, sterile inner layer and a loose outer layer that is colonized by commensal bacteria. Immunofluorescence microscopy of mouse and human colon tissues demonstrated that the SIgA co-localizes with gut bacteria in the outer mucus layer. Using mice genetically deficient for pIgR and/or mucin-2 (Muc2, the major glycoprotein of intestinal mucus), we found that Muc2 but not SIgA was necessary for excluding gut bacteria from the inner mucus layer in the colon. Our findings support a model whereby SIgA is anchored in the outer layer of colonic mucus through combined interactions with mucin proteins and gut bacteria, thus providing immune protection against pathogens while maintaining a mutually beneficial relationship with commensals.

## 1. Introduction

The human intestinal tract is home to some 100 trillion microorganisms, tenfold more than the total number of host cells throughout the body [1]. The mucosal immune system in the gut faces the daunting task of eliminating potential pathogens while maintaining a mutually beneficial relationship with the commensal microbiota. Antibodies of the secretory IgA (SIgA) class act as the first line of antigen-specific immunity in the gut, recognizing both pathogens and commensals [2,3]. Indeed, it has been estimated that up to 74% of bacteria in the gut lumen are coated with SIgA [4]. These SIgA antibodies contribute to adaptive immunity via specific antibody activity and to innate immunity via glycan-mediated binding to gut bacteria [5,6]. Together, these immune functions promote intestinal homeostasis by neutralizing potentially hazardous microbes, shaping the composition of the commensal microbiota, and preventing inappropriate inflammatory responses to microbial and food antigens [3].

IgA produced by local plasma cells in the mucosal *lamina propria* is transported into gut secretions by the polymeric immunoglobulin receptor (pIgR), a transmembrane glycoprotein produced exclusively by mucosal and glandular epithelial cells [7,8]. At the luminal surface, proteolytic cleavage releases the extracellular domain of pIgR, known as the secretory component (SC). In secretions, SC is present both in uncomplexed form and covalently bound to IgA as a part of the SIgA complex. SC enhances adaptive immunity by protecting IgA from degradation by host and microbial proteases in the harsh environment of the gut tract [9,10]. SC also contributes to innate immunity, by glycan-dependent adherence to bacteria and neutralization of pro-inflammatory host factors [11,12]. The critical roles of pIgR and SC in intestinal immunity is evidenced by the finding that mice with a targeted deletion of the *Pigr* gene have dramatically reduced IgA levels in gut secretions, are more susceptible to a range of intestinal infections, and have an altered composition of the commensal gut microbiota [7,13]. In humans, polymorphisms in the *Pigr* gene locus have been linked to increased susceptibility to inflammatory bowel diseases [14,15].

A landmark study published in 2002 demonstrated that SC ensures the appropriate localization of SIgA in the respiratory tract of mice, by anchoring IgA to the mucus gel lining the luminal surface [16]. These investigators subsequently demonstrated, using isolated loops of rabbit ileum, that monoclonal SIgA directed against the pathogenic bacterium *Shigella flexneri* preferentially associated with the mucus layer in the small intestine, and restricted *S. flexneri* to the luminal surface [17]. Less is known about the localization of SIgA in the colon, where the bacterial burden is dramatically higher and is largely comprised of resident commensals. It has been estimated that up to 3 g/day of SIgA is secreted into the gut of healthy humans [18,19], representing a potential availability of 10^7^ SIgA molecules for each of the 100 trillion resident bacteria [20].

The mucus layer is thicker in the intestinal tract than at other mucosal surfaces, comprising a dense inner layer (increasing in thickness from 15–30 µM in the small intestine to ~100 µM in the colon) and a loose outer layer (100–400 µM in the small intestine and ~700 µM in the colon) [21]. The critical role of mucus in intestinal immunity was highlighted by the finding that the dense inner mucus layer is devoid of bacteria [22]. The major glycoprotein constituent of intestinal mucus is mucin-2 (Muc-2) [23], the product of the Muc2 gene in humans and the Muc2 gene in mice, and secreted primarily by goblet cells. Mice with a targeted deletion in the Muc2 gene have reduced numbers of goblet cells and are completely devoid of a mucus layer at the luminal surface [24].

Here we used genetic and microscopic techniques to determine the localization of SIgA in colonic mucus, relative to the distribution of Muc2 protein and gut bacteria. Surprisingly, we found that the SIgA is relatively absent from the inner mucus layer, and is instead found to be associated with gut bacteria in the outer mucus layer. These findings have implications regarding the mechanisms through which SIgA provides immune protection in the colon, where its major function is to restrict entry of commensal bacteria into the body proper.

## 2. Results and Discussion

### 2.1. Colons of Mice that do not Express Mucin-2 Exhibit Abnormal Crypt Morphology and Absence of an Apical Mucus Layer

**Figure 1:**
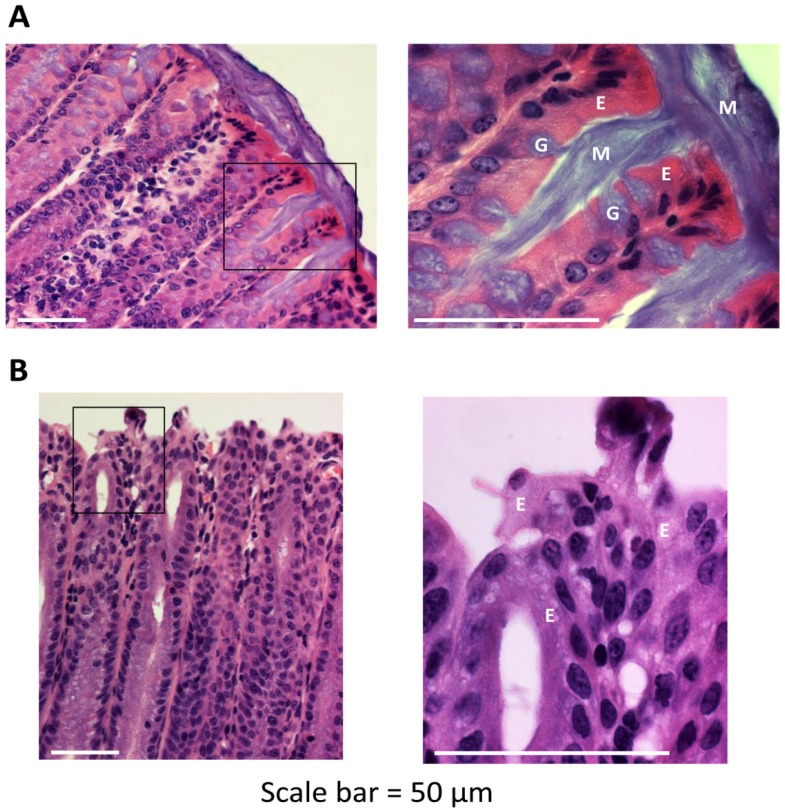
Architecture of the colonic mucosa in the presence or absence of mucin-2 (Muc2). Freshly dissected colons from 6–8 week-old wild-type (**A**) and *Muc2^−/−^* (**B**) mice were preserved in Carnoy’s fixative and whole mounted to preserve the integrity of the mucus layer. Sections of colon tissue were stained with hematoxylin and eosin. The areas within the black rectangles in the left-hand panels are magnified in the right-hand panels. Scale bar = 50 µM. E, enterocyte; G, goblet cell; M, mucus. Images are representative of multiple locations along the colon for at least three mice of each genotype.

To assess the importance of Muc2 protein in maintenance of normal architecture and function of the colonic mucosa, we compared histological images of colons from wild-type and *Muc2^−/−^* mice (Figure 1). Freshly dissected colons were preserved and whole mounted using a method that preserves the integrity of the fragile mucus layer [22]. Goblet cells were abundant in the colonic epithelium of wild-type mice, and could be observed extruding a thick mucus secretion that adhered to the luminal surface. By contrast, the epithelium of *Muc2^−/−^* mice was devoid of goblet cells and lacked a luminal mucus layer, consistent with published reports [24]. In addition, colons of *Muc2^−/−^* mice consistently exhibited crypt lengthening, an aberration often associated with chronic colitis [25]. However, we did not observe clinical signs of acute colitis in young *Muc2^−/−^* mice, such as weight loss, diarrhea or rectal bleeding. *Muc2^−/−^* mice have been reported to develop colitis spontaneously with age [26], although the penetrance of this phenotype is lower in *Muc2^−/−^* mice on the C57BL/6 genetic background than on certain other backgrounds, and is lessened by housing the mice in sterile cages with autoclaved food and water [27]. In summary, our findings are consistent with a critical role for Muc2 protein in establishment and maintenance of the mucus layer lining the colonic lumen.

### 2.2. Secretory IgA Localizes to the Outer Mucus Layer in Mouse Colonic Mucosa

**Figure 2:**
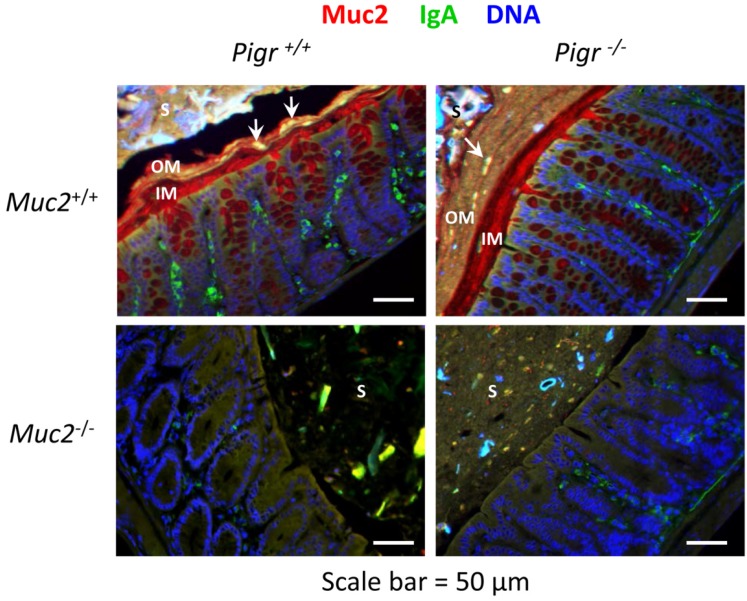
Localization of Muc2 and IgA in the colon in the presence and absence of the polymeric immunoglobulin receptor (pIgR) and mucin-2 (Muc2). Freshly dissected colons from 6–8 week-old *Muc2^+/+^Pigr^+/+^*, *Muc2^−/−^Pigr^+/+^*, *Muc2^+/+^Pigr^−/−^* and *Muc2^−/−^Pigr^‑/−^* mice were preserved in Carnoy’s fixative and whole mounted to preserve the integrity of the mucus layer. Sections of colon tissue were stained by immunofluorescence to visualize Muc2 (red) and IgA (green), then counterstained with DAPI (blue) to visualize cell nuclei. Scale bar = 50 µM. IM, inner mucus layer; OM, outer mucus layer; S, stool. Arrowheads denote co-localization of IgA and Muc2. Images are representative of multiple locations along the colon for at least three mice of each genotype. It should be noted that the thickness of the outer mucus layer can vary due to compression by adjacent stool material and/or stripping of the loose mucus during tissue processing.

Previous studies of the respiratory tract of mice have suggested that SIgA is anchored in the epithelial mucus layer through glycan-dependent associations of the SC moiety with mucin proteins [16]. We therefore hypothesized that the concentration of SIgA would be highest in the inner layer of colonic mucus, where it would contribute to the exclusion of bacteria. To test this hypothesis, we examined the localization of Muc2 and IgA by immunohistochemistry in colons of wild-type mice compared to mice with targeted deletions in the *Muc2* and/or *Pigr* genes (Figure 2). Surprisingly, the inner mucus layer in colons of wild-type mice was rich in Muc2 but relatively devoid of IgA. By contrast, Muc2 and IgA were frequently found to be co-localized in the outer mucus layer. IgA was also absent in the inner mucus layer of *Muc2^+/+^Pigr^−/−^* mice, consistent with the essential role for pIgR in epithelial transcytosis of IgA [28]. We occasionally observed co-localization of Muc2 and IgA in the outer mucus layer of *Muc2^+/+^Pigr^−/−^* mice, perhaps deriving from debris from shed epithelium containing IgA in the *lamina propria.* In colons of *Muc2^−/−^Pigr^+/+^* mice lacking a mucus layer, IgA could be seen in loose aggregates throughout the stool, often adjacent to the epithelial surface. However, IgA was rarely seen in the stools of *Muc2^−/−^Pigr^−/−^* mice.

**Figure 3:**
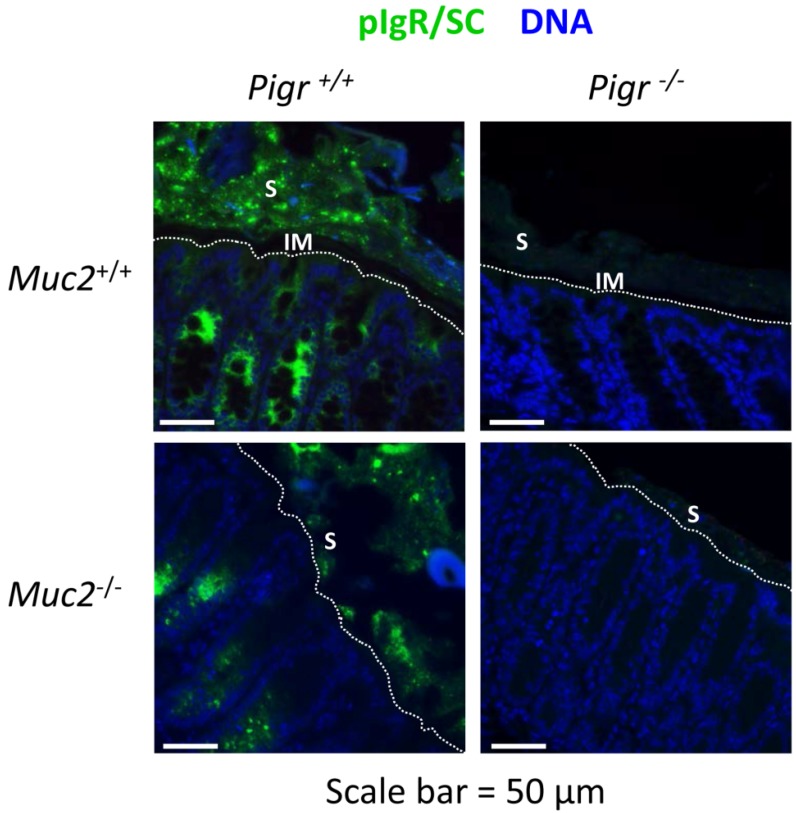
Localization of pIgR, secretory component (SC) and secretory IgA (SIgA) in the colon in the presence and absence of Muc2 and pIgR. Freshly dissected colons from 6–8 week-old *Muc2^+/+^Pigr^+/+^*, *Muc2^−/−^Pigr^+/+^*, *Muc2^+/+^Pigr^−/−^* and *Muc2^−/−^Pigr^‑/−^* mice were preserved in Carnoy’s fixative and whole mounted to preserve the integrity of the mucus layer. Sections of colon tissue were stained by immunofluorescence with an antibody to SC (green), the extracellular domain of pIgR. This antibody recognizes membrane-bound pIgR, free SC, and SIgA. Sections were counterstained with DAPI (blue) to visualize cell nuclei. Scale bar = 50 µM. White dotted lines indicate the boundary between the epithelial surface and the intestinal lumen. IM, inner mucus layer; S, stool. The IgA-devoid inner mucus layer in *Muc2^+/+^* mice is evidenced by the gap between the epithelial surface and IgA present in the outer mucus layer and stool. The boundary between the outer mucus layer and stool cannot be distinguished with this staining method. Images are representative of multiple locations along the colon for at least three mice of each genotype.

To examine further the localization of SIgA, sections of colon tissue were stained with an antibody to mouse SC, which recognizes membrane-bound pIgR, free SC and SIgA (Figure 3). In the colons of wild-type mice, a thin SC-devoid area above the epithelial surface was consistent with the absence of SIgA in the inner mucus layer. However, bright staining for SC was observed in the area above this “gap”, including adjacent stool material. Patches of SC were observed on the epithelial surface of colons from *Muc2^−/−^Pigr^+/+^* mice, despite the absence of a mucus layer. Patches of SC were observed in the stool and adjacent to the epithelial surface in colons from *Muc2^−/−^Pigr^+/+^* mice, similar to the staining pattern for IgA in Figure 2. As expected, no staining for SC was observed in colons of *Pigr^−/−^* mice, regardless of the presence or absence of Muc2. Our data suggest that Muc2 and pIgR act coordinately to localize SIgA in the outer mucus layer.

### 2.3. Gut Bacteria and SIgA Are Concentrated in the Outer Mucus Layer in Mouse Colonic Mucosa

**Figure 4:**
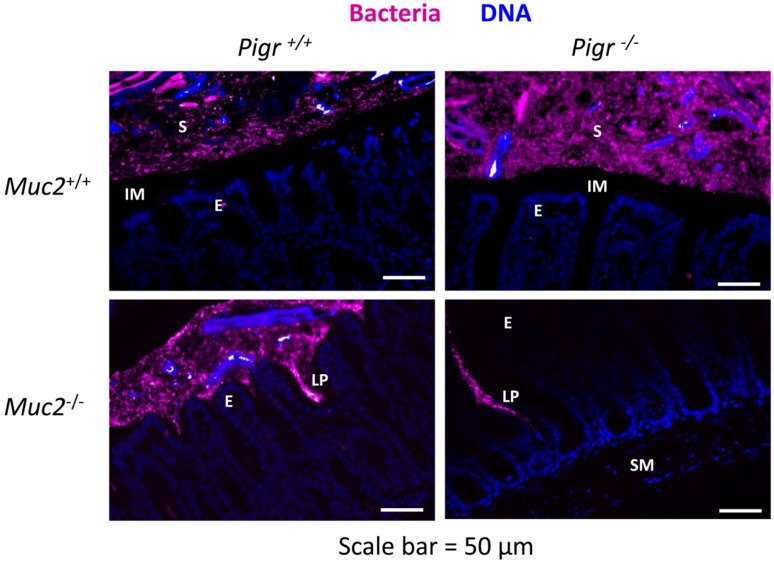
Invasion of bacteria into the colonic *lamina propria* in the absence of Muc2. Freshly dissected colons from 6–8 week-old *Muc2^+/+^Pigr^+/+^*, *Muc2^−/−^Pigr^+/+^*, *Muc2^+/+^Pigr^−/−^* and *Muc2^−/−^Pigr^‑/−^* mice were preserved in Carnoy’s fixative and whole mounted to preserve the integrity of the mucus layer. Sections of colon tissue were stained by fluorescence *in situ* hybridization for bacterial 16S rRNA (magenta), then counterstained with DAPI to visualize cell nuclei (blue). Scale bar = 50 µM. E, enterocytes at the luminal surface; IM, inner mucus layer; LP, *lamina propria*; S, stool; SM, sub-mucosa. The inner mucus layer in *Muc2^+/+^* mice is evidenced by the gap between the epithelial surface and bacteria present in the outer mucus layer and stool. Bacteria are in direct contact with the epithelial surface in *Muc2^−/−^* mice. The boundary between the outer mucus layer and stool cannot be distinguished with this staining method. Images are representative of multiple locations along the colon for at least three mice of each genotype.

Our data combining genetic and immunohistochemical approaches led to the surprising conclusion that SIgA localizes primarily in the outer layer of colonic mucus, rather than in the dense, Muc2-rich inner mucus layer (Figure 2 and Figure 3). Since the inner mucus layer has been shown to be relatively devoid of bacteria [22], we hypothesized that SIgA is anchored in the outer mucus layer through association with gut bacteria. To test this hypothesis, we used *in situ* fluorescence hybridization (FISH) of bacterial 16S rRNA to visualize the spatial relationship of gut bacteria with the epithelium in colons from wild-type mice and mice with targeted deletions in the *Muc2* and/or *Pigr* genes (Figure 4). In the colons of wild-type mice, a thin area above the epithelial surface was found to be devoid of bacteria, consistent with the relatively sterile nature of the Muc2-rich inner mucus layer. A similar bacteria-free zone was observed above the epithelial surface in colons from *Muc2^+/+^Pigr^−/−^* mice, suggesting that antimicrobial factors in the dense mucus gel were sufficient to exclude bacteria, even in the absence of SIgA. However, the absence of a mucus layer in *Muc2^−/−^* mice resulted in direct contact of gut bacteria with the epithelial surface, regardless of the presence or absence of pIgR. In these mice, bacterial invasion deep into the *lamina propria* was often observed, although we saw no evidence of bacteria in the submucosa. Although we did not directly measure antimicrobial activity, our findings are consistent with the concept that trapping of antimicrobial peptides in the dense inner mucus layer facilitates bacterial killing. In the absence of this mucus layer, antimicrobial peptides would be expected to diffuse more rapidly into the intestinal lumen, allowing bacteria to penetrate the epithelial barrier and gain access to the *lamina propria*.

Interestingly, we observed abundant DNA (visualized by blue staining in Figure 4) in the stools and/or the outer mucus layer of the colon. Sources of DNA could include shed epithelial cells (eukaryotic DNA) as well as intracellular and extracellular prokaryotic DNA. In this context it is important to note that self-organization of bacterial biofilms has been shown to be facilitated by extracellular DNA [29]. Several studies have suggested that SIgA stabilizes bacterial biofilms through dual interactions with intestinal mucin and bacterial cell surface proteins (reviewed in [30]). Our findings are consistent with a model in which complex interactions among SIgA, mucus, and extracellular DNA promote the formation of biofilms of commensal bacteria, which may contribute to protection of the epithelial surface.

**Figure 5:**
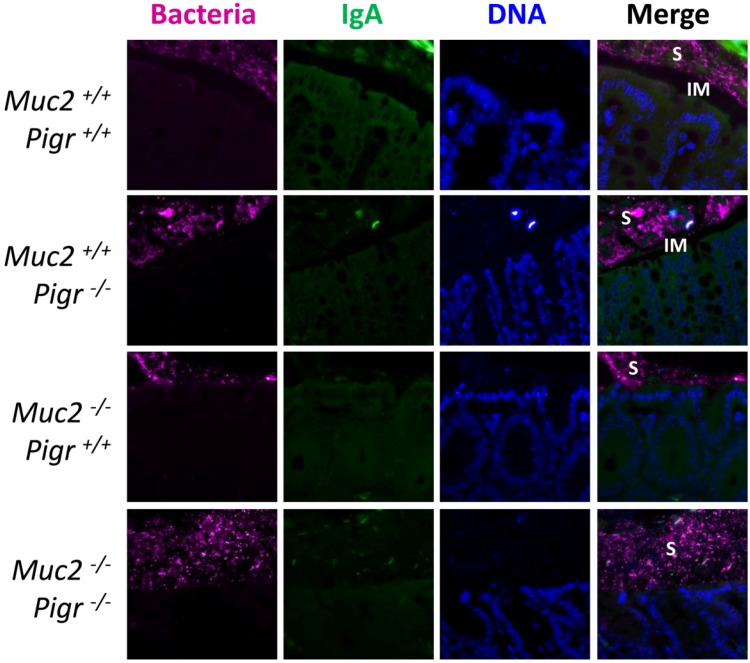
Localization of bacteria and IgA in the colon in the presence and absence of the pIgR and Muc2. Freshly dissected colons from 6–8 week-old *Muc2^+/+^Pigr^+/+^*, *Muc2^−/−^Pigr^+/+^*, *Muc2^+/+^Pigr^−/−^* and *Muc2^−/−^Pigr^‑/−^* mice were preserved in Carnoy’s fixative and whole mounted to preserve the integrity of the mucus layer. Sections of colon tissue were stained by fluorescence *in situ* hybridization for bacterial 16S rRNA (magenta), and by immunofluorescence for IgA (green), then counterstained with DAPI to visualize cell nuclei (blue). Scale bar = 50 µM. IM, inner mucus layer; S, stool. The IgA-devoid inner mucus layer in *Muc2^+/+^* mice is evidenced by the gap between the epithelial surface and IgA present in the outer mucus layer and stool. Bacteria are in direct contact with the epithelial surface in *Muc2^−/−^* mice. The boundary between the outer mucus layer and stool cannot be distinguished with this staining method. Images are representative of multiple locations along the colon for at least three mice of each genotype.

To determine whether SIgA was associated with gut bacteria in the presence or absence of a normal mucus layer, we combined FISH with immunostaining for IgA in colons from wild-type mice and mice with targeted deletions in the *Muc2* and/or *Pigr* genes (Figure 5). In wild-type mice, a zone adjacent to the epithelial surface, presumably the Muc2-rich inner mucus layer, was observed to be devoid of both bacteria and IgA. By contrast, both bacteria and IgA were observed in the stool (and likely also in the outer mucus layer, which cannot be distinguished from stool with this staining method). The absence of pIgR dramatically decreased the amount of IgA in stools of *Muc2^+/+^Pigr^−/−^* mice, but the bacteria-free zone adjacent to the epithelial surface was retained. However, as seen in Figure 4, large numbers of bacteria were observed at the epithelial surface of *Muc2^−/−^* mice, regardless of the presence or absence of pIgR.

### 2.4. Muc2 and IgA Co-Localize in the Outer Layer of Colonic Mucus in Humans

**Figure 6:**
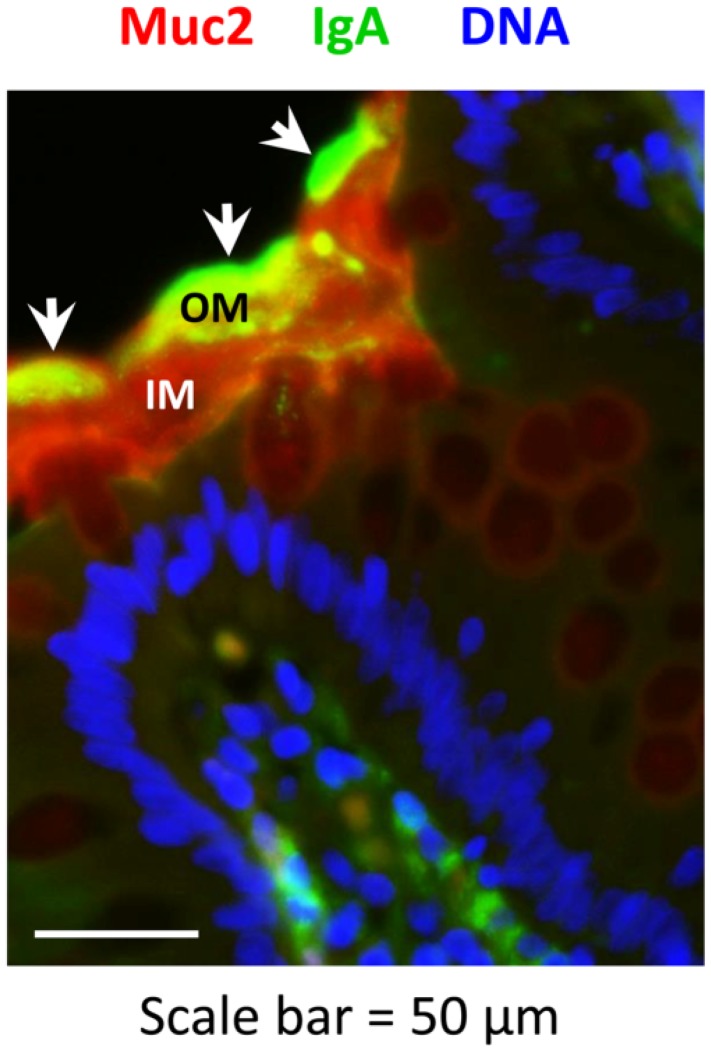
Localization of Muc2 and IgA in the human colon. Biopsies of healthy colonic mucosa from adult volunteers were obtained during routine colonoscopy. Sections of colon tissue were stained by immunofluorescence to visualize Muc2 (red) and IgA (green), then counterstained with DAPI (blue) to visualize cell nuclei. Scale bar = 50 µM. IM, inner mucus layer; OM, outer mucus layer. Arrowheads denote co-localization of IgA and Muc2. Images are representative of colonic biopsies from three healthy individuals.

Our imaging studies of mouse colons suggested that Muc2 and IgA co-localize in the outer mucus layer rather than in the inner layer. To determine whether this pattern holds true in humans, we obtained biopsies of colon tissue from healthy adult volunteers undergoing routine screening colonoscopy. Visualization of Muc2 and IgA by immunofluorescence revealed a pattern almost identical to that seen in mice: a dense Muc2-rich inner layer devoid of IgA, and a outer layer in which Muc2 and IgA were co-localized (Figure 6).

## 3. Experimental Section

### 3.1. Mice

Breeding pairs of wild-type C57BL/6J mice were obtained from Jackson Laboratories, Bar Harbor, ME. Breeding pairs of mice with a targeted mutation in the *Muc2* gene [24], backcrossed onto the C57BL/6 strain, were a kind gift from Dr. Anna Velcich, Albert Einstein College of Medicine of Yeshiva University, New York, NY, USA. Breeding pairs of mice with a targeted mutation in the *Pigr* gene [28], backcrossed onto the C57BL/6 strain, were obtained from the Mutant Mouse Regional Resource Center at the University of Missouri, Columbia, MO, USA (strain B6.129P2-*Pigr*^tm1Fejo^/Mmmh). *Muc2^−/−^Pigr^−/−^* double knockout mice were obtained by breeding *Muc2^−/−^* and *Pigr^−/−^* single knockout mice. All mice used for experiments were bred in-house. Mice were housed in sterilized microisolator cages in an AALAC accredited facility, and given autoclaved food and water. Procedures were conducted in compliance with the University of Kentucky Institutional Animal Care and Use Committee.

### 3.2. Collection of Colon Tissue Biopsies from Human Subjects

Adult volunteers were recruited from patients undergoing routine screening colonoscopy at the University of Kentucky Medical Center after institutional review board approval and written informed consent. Individuals were classified as normal when endoscopic, radiologic and pathologic evaluation of randomly obtained biopsies revealed no disease of the small or large bowel. Biopsied tissue was fixed in formalin and embedded in paraffin.

### 3.3. Harvesting and Processing of Colon Tissue

Freshly dissected mouse colons were collected without disturbing the luminal contents and fixed in Carnoy’s fixative (60% ethanol, 30% chloroform, 10% acetic acid) to preserve the mucus layer [22]. Fixed mouse and human colon tissues were embedded in paraffin, sectioned, de-waxed in xylene and rehydrated with ethanol gradients as described [31]. Sections were stained with hematoxylin and eosin to visualize tissue architecture or processed as described below for immunofluorescence or fluorescence *in situ* hybridization.

### 3.4. Immunofluorescence Microscopy

Antigen retrieval was performed by incubating tissue sections for 20 min at 95 °C in 10 mM sodium citrate, pH 6. Tissue sections were blocked by incubation at room temperature for 30 min in phosphate-buffered saline containing 0.1% Triton X-100 and 10% serum from the species used for the secondary antibody (see below). Where indicated, endogenous peroxidase activity was quenched by incubation at room temperature for 10 min in 3% (*v*/*v*) H_2_0_2_. Tissue sections were incubated at room temperature overnight with one or more of the indicated primary antibodies, diluted in blocking buffer: rabbit antibody to mouse or human Muc2, diluted 1:100 (Santa Cruz Biotechnology, Santa Cruz, CA, USA); goat antibody to mouse SC (the extracellular domain of pIgR), diluted 1:250 (R&D Systems, Minneapolis, MN, USA). Tissue sections were washed with phosphate-buffered saline, then incubated at room temperature for 2 h with one or more of the indicated secondary antibodies, diluted in blocking buffer: rhodamine-conjugated bovine antibody to rabbit IgG, diluted 1:500 (Santa Cruz Biotechnology); fluorescein-conjugated goat antibody to mouse or human IgA, diluted 1:200 (Santa Cruz Biotechnology); horseradish peroxidase-conjugated rabbit antibody to goat IgG, diluted 1:500 (Life Technologies, Grand Island, NY, USA). Where indicated, bound peroxidase was visualized with TSA Plus-TMR Reagent (Perkin-Elmer, Waltham, MA, USA). Tissue sections were counterstained with 4',6-diamidino-2-phenylindole (DAPI) to visualize eukaryotic cell nuclei. Imaging was performed on a Zeiss Axiophoto confocal microscope with Axiovision image software.

### 3.5. Fluorescence in Situ Hybridization for Visualization of Colonic Bacteria

After rehydration, tissue sections were hybridized with a universal 16S rRNA probe (EUB338) [22] at a concentration of 10 ng/µL as previously described [32]. The EUB338 probe, labeled on the 5’ end with AlexaFluor647 and purified by high performance liquid chromatography and agarose gel electrophoresis, was obtained from Life Technologies, Grand Island, NY. Tissue sections were counterstained with DAPI to visualize eukaryotic cell nuclei. Imaging was performed on a Zeiss Axiophoto confocal microscope with Axiovision image software.

## 4. Conclusions

The ability of SIgA to associate with the epithelial surface of the colonic mucosa is critical for its dual roles of excluding pathogens and maintaining a mutually beneficial relationship with commensals. Current models suggest that SIgA adheres to the mucus layer by association of glycan side chains of SC with the heavily glycosylated mucin proteins [11,16,17]. Our new findings suggest a more complex mechanism in the bacteria-rich environment in the colon. By comparing *Pigr^+/+^* and *Pigr^−/−^* mice, we found that the localization of commensal bacteria in the outer layer of colonic mucus was similar in the presence or absence of SIgA. Indeed, recent functional and metagenomic studies have identified a number of mucus-binding proteins expressed by commensal and probiotic bacteria (reviewed in [33]). The absence of bacteria in the inner layer of colonic mucus is likely due to the high local concentration of antimicrobial peptides (reviewed in [34,35,36]). We suggest that anchoring of SIgA in the outer layer of colonic mucus is enhanced through interactions of SIgA with bacteria, which could involve antigen-antibody binding via the IgA moiety as well as carbohydrate-dependent binding to the bacterial surface. In support of this model, a recent study demonstrated that the N-glycan side chains of SC mediate the association of SIgA with gram-positive commensals [6]. This model does not rule out the possibility of direct interactions between SIgA and mucin proteins, although the affinity of these interactions does not appear to be sufficient to retain SIgA in the inner mucus layer. In the outer mucus layer, weak interactions between SIgA and mucus may synergize with strong interactions between SIgA and gut bacteria. The current studies were limited to tissues from disease-free mice and healthy humans; we propose that future studies of the localization of SIgA during disease could shed light on the pathogenesis of inflammatory and infectious diseases of the colon.
